# Prevalence and Determinants of Generalized Anxiety Disorder Symptoms in Residents of Fort McMurray 12 Months Following the 2020 Flooding

**DOI:** 10.3389/fpsyt.2022.844907

**Published:** 2022-06-24

**Authors:** Ernest Owusu, Reham Shalaby, Ejemai Eboreime, Nnamdi Nkire, Mobolaji A. Lawal, Belinda Agyapong, Hannah Pazderka, Gloria Obuobi-Donkor, Medard K. Adu, Wanying Mao, Folajinmi Oluwasina, Vincent I. O. Agyapong

**Affiliations:** ^1^Department of Psychiatry, University of Alberta, Edmonton, AB, Canada; ^2^Global Psychological E-Health Foundation, Edmonton, AB, Canada; ^3^Department of Psychiatry, Dalhousie University, Halifax, NS, Canada

**Keywords:** anxiety, flooding, depression, disaster, traumatic

## Abstract

**Background:**

The flood in Fort McMurray (FMM) which occurred between April 26 and May 2, 2020, is known to have displaced an estimated population of 1,500 people, and destroyed or damaged about 1,230 buildings. In all, it is estimated to have caused about $228 million in losses.

**Objective:**

This study aims to identify the prevalence and determinants of likely Generalized Anxiety disorder (GAD) in among respondents 12-months after the 2020 flooding.

**Methods:**

Data for the study were collected through a cross-sectional survey sent through REDCap and hosted online from the 24th of April to the 2nd of June 2021. The self-administered questionnaire was emailed to respondents using community, government, school, and occupational platforms. Demographic, flooding-related variables, and clinical data were collected. A validated instrument, the GAD-7 was used to collect information on likely GAD. Consent was implied by completing the survey forms, and the University of Alberta Health Research Ethics Committee approved the study.

**Results:**

Of the 249 residents surveyed, 74.7% (186) respondents completed the online survey, 81.6% (80) were above 40 years, 71% (132) were in a relationship, 85.5% (159) were females, and 94.1% (175) were employed. The prevalence of likely GAD was 42.5% in our study. Predictors of likely GAD among respondents included positive employment status (OR = 30.70; 95% C.I. 2.183–423.093), prior diagnosis of depression (OR = 3.30; 95% C.I. 1.157–9.43), and the perceived need to have mental health counseling (OR = 6.28; 95% C.I. 2.553–15.45).

**Conclusion:**

This study showed that there was an increased magnitude of moderate to high anxiety symptoms among respondents following the natural disaster particularly the flood in 2020. The predictors of likely GAD include positive employment status, history of depression diagnosis, and the need to have mental health counseling. Policymakers may mitigate the rise of anxiety after flooding in vulnerable areas by addressing these and other factors.

## Introduction

Natural disasters like floods, earthquakes, tsunamis, and wildfires are known to cause havoc including disruption to life and damage to properties across affected countries and communities (1). Businesses, employment, housing, and lives are negatively impacted, causing survivors of such disasters to suffer financial, emotional, psychological, and physical health problems. Natural disasters particularly flooding could and usually do affect different populations, but a subset of those exposed usually suffer from symptoms of stress, and anxiety (2, 3) that are clinically significant and may require the intervention of mental health professionals (3).

Floods are considered the most frequent form of natural disaster and occur usually when there is an overflow of water that submerges land that is usually dry (4).

Evidence of the psycho-social effect of flood events suggests that flooding can pose substantial mental health and social problems that may continue over extended periods of time (5). Flooding can affect the psycho-social resilience of the hardiest of people who are affected by the flooding itself, or by secondary stressors that are indirectly related to the initial extreme event such as economic stress associated with re-building and that which arise as people try to recover their lives, property, and relationships (5).

The effects of flooding include homelessness, financial crisis, displacements, disruption to lives, shortages of foods and supplies, disruption of electricity or power supply, and potable water supply to the affected communities (6). Individuals exposed to these stressors commonly struggle with mental health challenges ranging from Generalized Anxiety Disorder GAD, substance use disorders, and depression (7).

The flooding in FMM in 2020 resulted in the displacement of an estimated 1,500 people and destroyed or damaged about 1,230 buildings between April 26 and May 2, 2020, and is estimated to have caused about $228 million in losses (6). Published research suggests that Generalized Anxiety Disorder (GAD) symptoms are increased following disasters, such as flooding (8, 9). As in other populations, we postulate that the flood has led to a rise in mental health disorders, particularly GAD (10) in FMM after the disaster. GAD is defined as “an uncontrollable disposition to worry about one's welfare and that of one's immediate kin” (11). The symptoms usually associated with GAD include arousal, vigilance, tension, irritability, unrestful sleep, and gastrointestinal distress (12).

The people of Fort McMurray have had to endure some traumas resulting from the 2016 wildfires, the COVID-19 pandemic and the attendants job losses and restrictions as well the flooding in 2020. These situations motivated the authors to carry out a study to estimate the level of trauma and the associated predictors caused by the flooding in 2020.

This study aims to estimate the prevalence and identify the determinants of likely GAD in residents of Fort McMurray (FMM) 12-months after the 2020 flooding.

## Methodology and Materials

### Setting and Design

This study was undertaken in Fort McMurray (FMM), an urban service area of the Northern Alberta Regional Municipality of Wood Buffalo in Canada. It is composed of a diverse population of ~111,687 as of the 2018 municipal population census (13). FMM is made of mainly temporary project accommodation facilities and has some surrounding rural communities (14).

The data for the study were collected through an online cross-sectional survey sent through REDCap (15). The REDCap is an online electronic platform for the capturing of data for research. The online survey link was sent to various electronic platforms, including the public and catholic school boards, the Canadian Mental Health Association, Keyano College and other community platforms from the 24th of April to the 2nd of June, 2021. The questionnaire was self-administered as respondents accessed them online. Consent was implied if the survey was completed, and the Alberta Health Research Ethics Committee approved the study.

### General Measure

The GAD-7 scale was used to assess the presence of anxiety symptoms amongst respondents. This scale consists of seven self-reported items that define the symptoms of GAD. Items on the scale are rated on a 4-point Likert-type scale. The responses for the questions were; Not at all (0), Several days (1), Over half the days (2), and Nearly every day (3). Scores ranged from 0 to 21 with higher scores indicating more severe symptoms of GAD (16). This study classified two types of anxiety; low anxiety is characterized by a score <10 and moderate to high anxiety (Likely anxiety) is characterized by scores of 10 or >10. The scale is known globally to show consistency and a one-factor structure in its usage (17) and shows that all its items are representative of one construct. The GAD-7 scale has been recommended as the most valid tool for assessing the severity of GAD symptoms in research and clinical practice (18, 19) Test-retest reliability was also found to be good (intraclass correlation = 0.83). Comparison of scores obtained from the self-report scales with those derived from the MHP-administered versions of the same scales yielded similar outcomes (intraclass correlation = 0.83), indicating good procedural validity. The internal consistency of the GAD-7 was excellent (Cronbach α = 0.92) (18, 19).

#### Sample Size Estimation

With a population of 111,687 as of the 2018 census (13), a 95% confidence interval, and a ±3% margin of error, the sample size needed for prevalence estimates for likely GAD will be 1,049. With an expected survey response rate of 20%, we planned to reach 5,290 residents with the online survey link.

### Collected Data and Statistical Analysis

A combination of socio-demographic factors (e.g., age, sex at birth, relationship status), clinical factors (e.g., clinical history of depression, anxiety), and flood-related variables (e.g., witnessing the flood or being fearful for one or others' lives) were collected through this survey. Statistical analysis for this study was completed using SPSS Version 25 (IBM 2011) (20). For demographic, clinical, and flood-related variables, descriptive statistics were used and centered on the respondents' age. Chi-square or Fisher exact (for small sample sizes) analysis was done to examine all the variables in association with the likely anxiety categorical variable (low and moderate to high anxiety). The significant (*p* ≤ 0.05) or near significant (0.1 > *p* > 0.05) variables obtained from the univariate analysis were included in a logistic regression model. Correlational analysis was used to rule out any strong inter-correlations (Spearman's correlation coefficient of 0.7 to 1.0 or −0.7 to −1.0) between the variables. The logistic regression model was employed to identify significant predictors of likely anxiety. Confidence intervals and odds ratios (OR) were used to determine the predictor variables, including wildfire-related factors for respondents to self-report likely anxiety after the 2016 wildfires in FMM. The reported data is devoid of imputations, representing the true and complete responses of respondents.

## Results

### Demographic Characteristics

Table 1 presents the demographic characteristics of the participants. In all, 186 out of the 249 residents who accessed the online survey completed it, giving a completion rate of 74.7%. Descriptive data of demographic, clinical, and flood-related data from the 186 respondents were analyzed against their age groupings as presented in Table 1. In all, 159 (85.5%) were females of which 80 (81.6%) were aged more than 40 years. Of the total respondents, 132 (71%) were in a relationship and 175 (94.1%) were employed with 50% of them employed by the school board. On the flood-related data, 176 (94.6%) of the respondents lived in FMM during the flooding, 31 (17.6%) lived in the flooding areas and respondents (*n* = 141, 75.8%), lived in their own homes before the flooding as against 45 (24.2%) who lived in rented homes. After the flooding, most respondents 154 (78.0%) live in their own homes whilst 41 (22.9%) live in rented homes. Regarding those who witnessed the flooding of homes and structures, 131 (75.3%) responded in affirmative and 51 (39.3%) were fearful for their lives and that of their friends and families.

**Table 1:** Demographic and flood-related variables by age of respondents.

**Variables**	**Age in years**		**Total, *n* (%)**
	**≤40 (*n*)**	**>40 (*n*)**	
**To which gender do you identify?**			
Male	9 (10.2%)	18 (18.4%)	27 (14.5%)
Female	79 (89.8%)	80 (81.6%)	159 (85.5%)
**Are you currently employed?**			
Yes	82 (93.2%)	93 (94.9%)	175 (94.1%)
No	6 (6.8%)	5 (5.1%)	11 (5.9%)
**If employed, where?**			
School boards	41 (50.6%)	46 (49.5%)	87 (50.0%)
Healthcare industry	5 (6.2%)	5 (5.4%)	10 (5.7%)
Keyano college	9 (11.1%)	11 (11.8%)	20 (11.1%)
Oil Sands industry	7 (8.6%)	6 (6.5%)	13 (7.5%)
Municipal or Government Agency	6 (7.4%)	7 (7.5%)	13 (7.5%)
Other	13 (16.0%)	18 (19.4%)	31 (17.8%)
**Marital status**			
In a relationship	62 (70.5%)	70 (71.4%)	132 (71.0%)
Not in a relationship	26 (29.5%)	28 (28.6%)	54 (29.9%)
**Did you reside at FMM during the 2020 flood?**			
No	7 (8.0%)	3 (3.1%)	10 (5.4%)
Yes		95 (96.9%)	176 (94.6%)
**Area of residence**			
No flooding areas	70 (86.4%)	75 (78.9%)	145 (82.4%)
Flooding areas	11 (13.6%)	20 (21.1%)	31 (17.6%)
**Where did you live prior to the 2020 FMM flooding?**			
Own home	61 (69.3%)	80 (81.6%)	141 (75.8%)
Renting	27 (30.7%)	18 (18.4%)	45 (24.2%)
**Where do you live now?**			
Own home	65 (73.9%)	80 (81.6%)	154 (78.0%)
Renting	23 (26.1%)	18 (18.4%)	41 (22.0%)
**Where did you live just prior to the 2020 FMM flooding?**			
In FMM	76 (95.0%)	91 (96.8%)	167 (96.0%)
Other	4 (5.0%)	3 (3.2%)	7 (4.0%)
**Did you witness the flooding of homes or structures in Fort MM?**			
No		23 (24.5%)	43 (24.7%)
Yes	60 (75.0%)	71 (75.5%)	131 (75.3%)
**Fearful of life and lives of family and friends during flood**			
No	55 (68.8%)	68 (72.3%)	123 (70.7%)
Yes	25 (31.3%)	26 (27.7%)	51 (29.3%)

Table 2 presents the clinical characteristics of the participants. With regards to a mental health diagnosis, 96 (51.6%) of all respondents have ever received a mental health diagnosis with a similar proportion within both age groups (49% of respondents aged = or <40 years and 48.0% of respondents aged > 40). Seventy-eight (41.9%) of all respondents were diagnosed specifically with Generalized Anxiety Disorder by a mental health professional. Within the age group = <40 years, 44.3% of them were diagnosed with GAD compared to 39.8% of respondents aged >40 years.

**Table 2:** Clinical characteristics by age of respondents.

**Variables**	**Age in years**		**Total, *n* (%)**
	**≤40 (*n*)**	**>40 (*n*)**	
**Prior history of depression diagnosis from a mental health professional**			
No	27 (30.7%)	31 (31.6%)	58 (31.2%)
Yes	61 (69.3%)	67 (68.4%)	128 (68.8%)
**Prior history of Bipolar diagnosis from a mental health professional**			
No	86 (97.7%)	94 (95.9%)	180 (96.8%)
Yes	2 (2.3%)	4 (4.1%)	6 (3.2%)
**Prior history of anxiety diagnosis from a mental health professional**			
No	49 (55.7%)	59 (60.2%)	108 (58.1%)
Yes	39 (44.3%)	39 (39.8%)	78 (41.9%)
**Prior history of Schizophrenia diagnosis from a mental**			
**health professional**			
No	88 (100.0%)	98 (100.0%)	186 (100.0%)
**Prior history of personality disorder diagnosis from a mental**			
**health professional**			
No	87 (98.9%)	97 (99.0%)	184 (98.9%)
Yes	1 (1.1%)	1 (1.0%)	2 (1.1%)
**Prior history of other mental health diagnosis from a health professional**			
No	78 (88.6%)	91 (92.9%)	169 (90.9%)
Yes	10 (11.4%)	7 (7.1%)	17 (9.1%)
**Prior history of mental health diagnosis (if any) from a**			
**health professional**			
No	39 (44.3%)	51 (52.0%)	90 (48.4%)
Yes	49 (55.7%)	47 (48.0%)	96 (51.6%)
**Prior history of Antidepressants medication use**			
No	61 (69.3%)	66 (67.3%)	127 (68.3%)
Yes	27 (30.7%)	32 (32.7%)	59 (31.7%)
**Prior history of antipsychotic medication use**			
No	86 (97.7%)	96 (98.0%)	182 (97.8%)
Yes	2 (2.3%)	2 (2.0%)	4 (2.2%)
**Prior history of benzodiazepines use**			
No	85 (96.6%)	97 (99.0%)	182 (97.8%)
Yes	3 (3.4%)	1 (1.0%)	4 (2.2%)
**Prior history of mood stabilizers use**			
No	83 (94.3%)	91 (92.9%)	174 (93.5%)
Yes	5 (5.7%)	7 (7.1%)	12 (6.5%)
**Prior history of sleeping tablets use**			
No	79 (89.8%)	86 (87.8%)	155 (88.7%)
Yes	9 (10.2%)	12 (12.2%)	21 (11.3%)
**Are you on any medication for a mental health concern?**			
No	87 (98.8%)	96 (98.0%)	183 (98.4%)
Yes	1 (1.1%)	2 (2.0%)	3 (1.6%)
**Are you on any of the following medication for a mental health concern?**			
Not on MH Rx	57 (64.8%)	63 (64.3%)	120 (64.5%)
Yes on MH Rx	31 (35.2%)	35 (35.7%)	66 (35.5)
**Have you received mental health counseling in the past?**			
No	46 (52.3%)	68 (69.4%)	114 (61.3%)
Yes	42 (47.7%)	30 (30.6%)	72 (38.7%)
**Would you like to receive mental health counseling?**			
No	33 (37.5%)	55 (56.1%)	88 (47.3%)
Yes	55 (62.5%)	43 (43.9%)	98 (52.7%)

Other diagnoses by a mental health professional reported by respondents included depression (68.8%), bipolar disorder (3.2%), personality disorders (1.1%), and none of the respondents reported a diagnosis of schizophrenia (0.0%). Sixty-six, 35.5% of total respondents were receiving medications for mental health concerns with 31.7% on antidepressants, 2.2% on antipsychotic, 2.2% on benzodiazepine, 6.5% on a mood stabilizer, and 11.3% on sleeping pills. More than half (61.3%) of the respondents had not received any mental health counseling even though 98 (52.1%) of the respondents were willing to receive mental health counseling.

### Source of Information and Support for Participants by Age Category

About 85% of the total respondents were monitoring the news and images relating to the floods at least daily on TV (66.7% watched daily and 21.3% watched more than daily) and about 96% read newspapers or internet-related articles at least daily (76.3% read daily and 20.2% read more than daily). Some respondents, 100 (59.9%) received some high level of support from the family after the flooding, 19 (11.1%) from the Red Cross, and 18 (10.5%) from the government of Alberta (Table 3).

**Table 3:** Source of information, degree of damage and support for participants.

**Variables**	**Age in years**		**Total, *n* (%)**
	**≤40 (*n*)**	**>40 (*n*)**	
**During the 2020 FMM flooding, how frequently did you watch television**			
**television images about the devastation caused by the flood?**			
Daily	46 (57.5%)	70 (74.5%)	116 (66.7%)
>Daily	20 (25.0%)	17 (18.1%)	37 (21.3%)
Did not watch at all	14 (17.5%)	7 (7.4%)	21 (12.1%)
**During the 2020 FMM flooding, how frequently did you read newspaper or**			
**internet articles related to the devastation caused by the flooding?**			
Daily	62 (77.5%)	70 (75.3%)	132 (76.3%)
>Daily	16 (20.0)	19 (20.4%)	36 (20.2%)
Did not read at all	2 (2.5%)	4 (4.3%)	6 (3.5%)
**Did you lose property as a result of the floods in FMM?**			
**Home was completely destroyed**			
Unchecked	88 (100.0%)	98 (100.0%)	186 (100.0%)
Checked	0 (0.0%)	0 (0.0%)	0 (0.0%)
**Home suffered substantial damages**			
Unchecked	86 (97.7%)	91 (92.9%)	177 (95.2%)
Checked	2 (2.3%)	7 (7.1%)	9 (4.8%)
**Home was slightly damaged**			
Unchecked	86 (97.7%)	95 (96.9%)	181 (97.3%)
Checked	2 (2.3%)	3 (3.1%)	5 (2.7%)
**Car was completely destroyed**			
Unchecked	88 (100.0%)	94 (95.9%)	182 (97.8%)
Checked	0 (0.0%)	4 (4.1%)	4 (2.2%)
**Business was completely destroyed by the floods**			
Unchecked	85 (96.6%)	95 (96.9%)	180 (96.8%)
Checked	3 (3.4%)	3 (3.1%)	6 (3.2%)
**Suffered no loss of property in the floods**			
No loss	81 (92.0%)	84 (85.7%)	165 (88.7%)
Yes loss	7 (8.0%)	14 (14.3%)	21 (11.3%)
**Did you live in the same house you lived in before the floods?**			
Yes	68 (86.1%)	79 (84.0%)	147 (85.0%)
No even though my home was not affected	10 (12.7%)	12 (12.8%)	22 (12.7%)
No because my home was affected	1 (1.3%)	3 (3.2%)	4 (2.3%)
**Did you receive family support during or after the floods?**			
Some to high level of support	51 (65.4%)	49 (55.1%)	67 (40.1%)
Limited or no support	27 (34.6%)	40 (44.9%)	100 (59.9%)
**Did you receive support from the Red Cross during or after the floods?**			
Some to high level of support	6 (7.6%)	13 (14.1%)	19 (11.1%)
Limited or no support	4 (5.1%)	13 (14.1%)	17 (9.9%)
Not impacted by the floods	69 (87.3%)	66 (71.7%)	135 (78.9%)
**Did you receive support from the Government of Alberta during or**			
**after the floods?**			
Some to high level of support	6 (7.6%)	12 (13.0%)	18 (10.5%)
Limited or no support	4 (5.1%)	14 (15.2%)	18 (10.5%)
Not Impacted by the floods	69 (87.3%)	66 (71.7)	135 (78.9%)
**Did you receive insurers support during or after the floods?**			
Some to high level of support	4 (5.1%)	6 (6.5%)	10 (5.8%)
Limited or no support	4 (5.1%)	14 (15.2)	18 (10.5%)
Not impacted by the floods	71 (89.9%)	7 (78.3%)	143 (83.6%)

### Association Between Demographic, Clinical, Flooding-Related Variables and General Anxiety Disorders

The univariate analysis of Table 4 is made of 23 demographics, clinical, and flooding related variables and their association with likely GAD. Chi-squared and Fisher exact test showed a significant association between likely GAD and nine variables: employment status, prior history of having been diagnosed with depression, prior history of anxiety disorder, prior history of any mental disorder diagnosis, taking antidepressants as well as taking benzodiazepine medication, taking no medication for a mental health concern, received mental health counseling, will like to receive a mental health counseling, fear of life and lives of friends and families during the flooding.

**Table 4:** Chi-squared test of association between demographic, clinical, flood-related variables and likely GAD.

**Variables**	**Low anxiety**	**Likely GAD**	**Chi-square**	***p*-Value**
**Gender**				
Male	15 (65.2%)	8 (34.8%)	0.653	0.499
Female	81 (56.3%)	63 (43.8%)		
**Age (years)**				
<40	40 (51.9%)	37 (48.1%)	1.792	0.210
≥40	56 (62.2%)	34 (37.8%)		
**Employment status**				
Unemployed	1 (11.1%)	8 (88.9%)	8.371	0.005
Employed	95 (60.1%)	63 (39.9%)		
**Place of employment**				
School boards	50 (66.7%)	25 (33.3%)	8.290	0.142
Healthcare industry	5 (55.6%)	4 (44.4%)		
Keyano college	10 (50.0%)	10 (50.0%)		
Oil Sands industry	6 (50.0%)	6 (50.0%)		
Municipal or Government Agency	10 (83.3%)	2 (16.7%)		
Other	50 (66.7%)	25 (33.3%)		
**Marital status**				
In a relationship	73 (60.3%)	48 (39.7%)	1.455	0.293
Not in a relationship	23 (50.0%)	23 (50.0%)		
**Did you reside in FMM during the 2020 flood?**				
No	4 (66.7%)	2 (33.3%)	0.215	0.703
Yes	92 (57.1%)	69 (42.9%)		
**Housing status prior to the flooding**				
Own home	78 (60.5%)	51 (39.5%)	2.060	0.191
Renting	18 (47.4%)	20 (52.6%)		
**Current housing status**				
Own home	78 (58.6%)	55 (41.4%)	0.361	0.565
Renting	18 (52.9%)	16 (47.1%)		
**Prior history of depression**				
Yes	19 (35.8%)	34 (64.2%)	14.871	0.000
No	77 (67.5%)	37 (32.5%)		
**Prior history of bipolar disorder**				
Yes	3 (60.0%)	2 (40.0%)	0.013	1.00
No	93 (57.4%)	69 (42.6)		
**Prior history of generalized anxiety disorder**				
Yes	29 (41.4%)	41 (58.6%)	12.713	0.000
No	67 (69.1%)	30 (30.9%)		
**Prior history of personality disorder**				
Yes	0 (0.0%)	100 (100.0%)	1.360	0.425
No	96 (57.8%)	70 (42.2%)		
**Prior history of a mental health diagnosis**				
Yes	41 (47.7%)	45 (52.3%)	6.983	0.012
No	55 (67.9%)	26 (32.1%)		
**Received antidepressants before the flood**				
Yes	24 (45.3%)	29 (54.7%)	4.730	0.043
No	72 (63.2%)	42 (36.8%)		
**Received anti-psychotics before the flood**				
Yes	2 (66.7%)	1 (33.3%)	0.105	1.000
No	94 (57.3%)	70 (42.7%)		
**Received Benzodiazepine before the flood**				
Yes	0 (0.0%)	4 (100.0%)	5.541	0.031
No	96 (58.9%)	67 (41.1%)		
**Received mood stabilizers before the flood**				
Yes	5 (50.0%)	5 (50.0%)	0.244	0.745
No	91 (58.0%)	66 (42.0%)		
**Received sleeping tablets before the flood**				
Yes	8 (44.4%)	10 (55.6%)	1.404	0.313
No	88 (59.1%)	61 (40.0%)		
**No medication for mental health concern**				
Yes	28 (48.7%)	32 (53.3%)	4.484	0.050
No	68 (63.6%)	39 (36.4%)		
**Receive MH counseling in the past**				
Yes	23 (36.5%)	40 (63.5%)	18.215	0.000
No	73 (70.2%)	31 (29.8%)		
**Would like MH counseling**				
Yes	31 (35.2%)	57 (64.8%)	37.708	0.000
No	65 (82.3%)	14 (17.7%)		
**Place of residence just prior to the McMurray 2020 flooding**				
In Fort MM	92 (56.5%)	70 (43.6%)	1.701	0.242
Other	5 (83.3%)	1 (16.7%)		
**Witnessed flooding of homes**				
No	23 (56.1%)	18 (43.9%)	0.043	0.857
Yes	73 (57.9%)	53 (42.1%)		
**Fearful of life and lives of family and friends during flood**				
No	75 (63.6%)	43 (36.4%)	6.072	0.016
Yes	21 (42.9%)	28 (57.1%)		

### Association Between Participants' Source of Information, Degree of Damage, Support, and General Anxiety Disorders

Table 5 shows chi-squared and Fisher exact analyses of participants' source of information, source of support for participants, and their association with likely GAD. The frequency of watching TV and images during the flood was found to be significantly associated with GAD symptoms among the study participants. Also, there was a significant association between GAD symptoms and the frequency of reading internet-related articles. No significant relationship was found between variables related to loss of property due to the flood, source of support, and likely GAD.

**Table 5:** Association between participants' source of information, degree of damage, support and likely GAD.

**Variables**	**Low anxiety**	**Likely GAD**	**Chi-square**	***p*-Value**
**Frequency watching TV images of the flooding**				
Daily	55 (50.0%)	55 (50.0%)	7.765	0.022
< Daily	27 (75.0%)	9 (25.0%)		
Did not watch	14 (66.7%)	7 (33.3%)		
**Frequency reading newspapers about the flood**				
Daily	66 (52.4%)	60 (47.6%)	7.765	0.022
< Daily	25 (73.5%)	9 (26.5%)		
Did not watch	4 (66.7%)	2 (33.3%)		
**Frequency reading newspapers about the flood**				
Daily	66 (52.4%)	66 (52.4%)	5.119	0.074
< Daily	25 (73.5%)	25 (73.5%)		
Did not read	4 (66.7%)	4 (66.7%)		
**Lose property due to the flood:**				
**Home suffered substantial damage**				
Unchecked	93 (58.9%)	65 (41.1%)	2.270	0.171
Checked	3 (33.3%)	6 (66.7%)		
**Home suffered slight damage**				
Unchecked	94 (58.0%)	68 (42.0%)	0.645	0.652
Checked	2 (40.0%)	3 (60.0%)		
**Car was completely destroyed**				
Unchecked	93 (57.1%)	70 (42.9%)	0.514	0.657
Checked	3 (75.0%)	1 (25.0%)		
**Business was completely destroyed**				
Unchecked	94 (58.0%)	68 (42.0%)	0.645	0.642
Checked	2 (40.0%)	3 (60.0%)		
**Suffered no loss of property**				
No loss	88 (59.9%)	59 (40.1%)	2.842	0.147
Yes loss	9 (40.0%)	12 (60.0%)		
**Live in same house prior to the flood**				
Yes	84 (59.6%)	57 (40.4%)	2.174	0.357
No (although home was not destroyed by flood)	9 (42.9%)	12 (57.1%)		
No (home destroyed by flood)	2 (50.0%)	2 (50.0%)		
**Family support after the flood**				
Some to high level of support	56 (57.7%)	41 (42.3%)	0.074	0.870
Limited support or no support	35 (55.6%)	28 (44.4%)		
**Support from Red Cross after the flood**				
Some to high level support	9 (52.9%)	8 (47.1%)	1.059	0.631
Limited or no support	8 (47.1%)	9 (52.9%)		
NA (Not impacted)	77 (59.2%)	53 (40.8%)		
**Support from Government of Alberta after the flood**				
Some to high level of support	7 (41.2%)	10 (58.8)	2.021	0.365
Limited or no support	10 (58.8%)	7 (41.2%)		
NA (Not impacted by flood)	77 (59.2%)	53 (40.8%)		
**Support from insurers during/after flood**				
Some to high level of support	5 (50.0%)	5 (50.0%)	2.390	0.310
Limited or no support	7 (41.2%)	10 (58.8%)		
NA (Not impacted by flood)	82 (59.9%)	55 (40.1%)		

### The Model Predicts Likely Anxiety Among Respondents

Illustrated in Table 6 below is the multivariate logistic regression model that was employed to predict likely GAD among respondents. The model included nine out of the 12 chi-square predictor variables. This was after removing three variables' *prior history of mental health diagnosis*, taking Benzodiazepines, and *prior history of receiving mental health counseling* which showed a high correlation with some other variables (*r*_s_ > 0.7).

**Table 6:** Multivariate logistic regression model for respondents' likelihood to present with moderate to severe anxiety.

**Characteristics**	***B***	**SE**	**Wald**	**df**	***p*-Value**	**Odd's ratio**	**95% C.I. for odd's ratio**	
							**Lower**	**Upper**
Employment status	3.425	1.349	6.444	1	0.011^*^	30.709	2.183	423.093
Prior history of depression	1.195	0.535	4.985	1	0.026^*^	3.303	1.157	9.429
Prior history of generalized anxiety disorder	0.446	0.557	0.643	1	0.423	1.563	0.525	4.651
Received Antidepressants before the flood	−0.631	0.607	1.078	1	0.299	0.532	0.162	1.750
Received Mental Health counseling in the past	0.220	0.493	0.198	1	0.656	1.245	0.474	3.273
Would like to receive a Mental Health counseling	1.837	0.459	16.000	1	0.000^*^	6.280	2.553	15.450
Fearful of life and lives of family and friends during flood	0.451	0.440	1.053	1	0.305	1.571	0.663	3.720
Frequency of watching TV images of the flood
Daily			3.389	2	0.184			
Less than daily	−1.371	0.770	3.163	1	0.075	0.254	0.056	1.149
Did not watch	−0.710	0.723	0.962	1	0.327	0.492	0.119	2.030
Frequency of reading internet related articles about flooding
Daily			0.099	2	0.052			
Less than daily	−0.191	0.790	0.059	1	0.809	0.826	0.175	3.887
Did not read	−0.289	1.174	0.061	1	0.806	0.749	0.075	7.482
Constant	−1.668	0.385	18.804	1	0.000	0.189		

*Statistically significant predictors at p ≤ 0.05.

The logistic regression model was statistically significant; *X*^2^ (df = 12; *n* = 161) = 75.39, *p* < 0.001, indicating that the model could differentiate between respondents who could or could not exhibit any likely anxiety during the 2020 FMM flooding. The model could account for 37.4% (Cox and Snell *R*^2^) to 50.1% (Nagelkerke *R*^2^) of the variance. Using the Hosmer–Lemeshow goodness-of-fit test, the model was adequately fit (χ^2^ = 10.0; *p* = 0.27) and correctly classified 75.8% of cases according to the goodness-of-fit statistic.

Three variables, namely *positive employment status* (OR = 30.70; 95% C.I. 2.183–423.093), *prior history of depression diagnosis* (OR = 3.30; 95% C.I. 1.157–9.43) and *will like to receive mental health counseling* (OR = 6.28; 95% C.I. 2.553–15.45) were the only independent significant predictors of likely GAD in the model. Employed respondents were 30 times less likely to present with likely GAD, with all other variables in the model controlled for.

Also, respondents with a history of depression were three times more likely to meet the criteria for likely GAD as opposed to those without that history.

It was also seen from the study that respondents who were willing to receive any mental health counseling were six times more likely to present with GAD symptoms.

## Discussion

The prevalence of likely GAD was 42.5% in our study. This prevalence was much higher in comparison to similar studies conducted 6 and 18 months after the wildfires in 2016 in the same community (20 and 18%, respectively) (7). Plausibly due to the huge impact of the flooding on the general wellbeing of the people within the setting of this study. Additionally, this study was carried out at a time when the people of FMM may not have fully recovered from the effects of the wildfires in 2016 when the flooding occurred. Furthermore, the effects of the COVID-19 pandemic and its attendant restrictions and job losses were still at play when the study was conducted.

Three key variables independently predicted likely GAD in this study when all variables were controlled for. These were positive employment status, prior history of depression, and the perceived need for mental health counseling. Respondents who were gainfully employed were 30 times less likely to present with likely GAD compared to their unemployed counterparts. This particular study outcome or finding is consistent with previous studies which reported a link between losing a job or job insecurity to various mental health conditions including but not limited to GAD, and psychological distress (21). According to a cohort study conducted in Australia, people who had job insecurity were found to be four times more likely to express severe psychological distress compared to those employed during the pandemic (22). During the periods of crippling economic activities, many individuals report being at risk of inability to cope with unexpected expenses, paying of ordinary bills and buying food (23). This has the likely tendency to impact negatively on mental health and increase the susceptibility to mental health problems, particularly GAD symptoms (24).

The present study also established that underlying depression diagnosis independently predicted the presence of likely GAD. Those who reported having a diagnosis of depression at the time of the survey were three times more likely to present with moderate to high anxiety symptoms. This is in concordance with previous literature, in which people who had previously established mental health conditions were found to be at a higher risk of developing mental illness post-disaster (17, 25). Similarly, a study by McPherson et al. (26) reported that preexisting mental health conditions were a key determining factor for psychological distress early in the study phase, but the effect was weakened later in the study. The possible explanation for this observation is that, people who have a well-established diagnosis of depression may be in a poorer position to deal with the stress and build resilience toward the post-disaster crisis than those without a preexisting depression diagnosis.

The willingness to receive any mental health counseling was also found to be a determinant for the likelihood of GAD. Respondents who reported a need for mental health counseling were found to be six times more likely to develop symptoms of GAD than those who were not willing to. The desire to have any mental health counseling communicates a psychological need (27, 28). This finding is in agreement with earlier studies that identifies psychological distress as a need to be addressed (28, 29). Another study established that, problems relating to hyperactivity were found to be reduced significantly after the application of the required intervention techniques including counseling in individuals who go through any form of crisis (30). Furthermore, understanding the importance of one's own existence and fixing and nurturing one's own self helped the survivors of disasters to enhance the quality of their wellbeing (30, 31). Other researchers also examined the emotional domain as a protective factor (30).

### Limitations

The relatively small sample size may be considered a limitation to generalizing inferences from this study. Whereas a more representative sample would have been desirous, in practice this was not possible given the limitations to direct data collection occasioned by the COVID-19 pandemic and attendant restrictions. Our approach of reaching the residents by email *via* intermediaries such as government, employers, schools, and community organizations was pragmatic, and ethically approved. We were, thus unable to report response rates but completion rates. Further, our findings should be interpreted with caution as it may more appropriately be reflective of Fort McMurray residents accessible *via* these social and occupational intermediaries, rather than the general population of the city. Additionally, this paper is focused on determining only the effects of the flooding on the anxiety level of the respondents and did not include the possible impact of the trauma caused by the prevailing COVID-19 pandemic as a contributory factor to the increased anxiety in respondents. The self-report nature of the likely GAD measurement scale takes away some objectivity and possible clinical assessment of the data provided by the respondents. Notwithstanding the limitations stated above, this study still adds to the limited literature of the predictors of GAD symptoms after natural or man-made disasters.

## Conclusion

The study established the prevalence and determinants of GAD symptoms among the residents of FMM 1 year after the 2020 flooding. Positive employment status, history of depression diagnosis, and a need to have mental health counseling were found to independently predict likely GAD. Policymakers need to put the necessary policies in place to ameliorate the suffering of the survivors after disasters. Counseling, as well as psychological and other mental health services should be made readily available to serve as support for survivors. It is believed that increasing the awareness of the people's perspective in managing flood risks will not only improve the effectiveness of the flood resilience-building process but also provide simultaneous benefits for social systems coping with the potential threat posed by climate extremes and climate change. Therefore, governments and provincial leaders as well as the local people should collaborate with mental health experts in managing pre and post-disaster challenges. Post-disaster, governments can implement population level interventions such as supportive text messaging programs, which have been found to be evidence based, easily scalable and cost effective (32–38). Finally, people with pre-existing mental health conditions should be considered as part of the vulnerable population and access to care and support services should be made available to them in order to help them cope with trauma that comes with any forms of disasters.

## Data Availability Statement

The raw data supporting the conclusions of this article will be made available by the authors, without undue reservation.

## Ethics Statement

The studies involving human participants were reviewed and approved by Alberta Health Research Ethics Committee. The patients/participants provided their written informed consent to participate in this study.

## Author Contributions

The study was conceived and designed by VA. RS performed the analysis and EO put together the initial manuscript. EE was involved in data collection. All other authors made significant contributions in reviewing and revising the final manuscript.

## Funding

Mental Health Foundation and the Douglas Harden Trust Fund provided the needed grants to support this project.

## Conflict of Interest

The authors declare that the research was conducted in the absence of any commercial or financial relationships that could be construed as a potential conflict of interest.

## Publisher's Note

All claims expressed in this article are solely those of the authors and do not necessarily represent those of their affiliated organizations, or those of the publisher, the editors and the reviewers. Any product that may be evaluated in this article, or claim that may be made by its manufacturer, is not guaranteed or endorsed by the publisher.
