# Supplementary material for: Prevalence and Determinants of Generalized Anxiety Disorder Symptoms in Residents of Fort McMurray 12 Months Following the 2020 Flooding
Source: Front Psychiatry. 2022 Jun 24;13:844907. doi: 10.3389/fpsyt.2022.844907 (PMC9263447; doi:10.3389/fpsyt.2022.844907)
Supplement: Supplementary file 1 [file Data_Sheet_1.PDF]

Fort McMurray Trauma Study

Please complete the survey below.

Thank you!

Information Leaflet

Fort McMurray Trauma Study

Principal Investigator: Dr. Vincent Agyapong 780-215-7771

You are being invited to take part in a clinical research study to be carried out in Fort McMurray.

Before you decide whether or not you wish to take part, you should read the information provided attached carefully.

Free FMMStrong Supportive Text Messaging Program

After the survey, you will be provided with an opportunity to enroll in the FMMStrong Supportive Text Message Program by entering your phone number in the space provided.

Once you are enrolled, you will receive 12 months of FREE daily mental support delivered through text messages to your mobile phone.

[Attachment: "Fort McMurray Trauma Study 2021 Information Leaflet.pdf"]

Baseline demographic and clinical information

1 To which gender identity do you most identify?
- ◯ Female
- ◯ Male
- ◯ Other

If other gender, please specify

____

2 Age (Years)

____

3 Are you currently employed?
- ◯ Yes
- ◯ No

Which of these categories do you best fit into?
- ◯ Stay at Home Parent
- ◯ Unemployed on social assistance
- ◯ Unemployed. No income
- ◯ Retired
- ◯ Student
- ◯ Other

If other, please specify

____

	If employed, where?	◯ School Boards<br>◯ Healthcare industry<br>◯ Keyano College<br>◯ Oil Sands Industry<br>◯ Municipal or Government Agency<br>◯ Other
	Please select the school board as applicable	◯ Catholic School Board<br>◯ Public School Board
	If other, please specify	__________________________________
4	Marital status	◯ Single<br>◯ Married<br>◯ Partnered/cohabiting<br>◯ Divorced/ separated<br>◯ Widowed
5	Did you reside at Fort MacMurray during the 2016 wildfire?	◯ Yes<br>◯ No
	Where did you reside during the 2016 wildfire?	__________________________________
	Area of residence during the 2016 wildfire	◯ Abasand<br>◯ Beacon Hill<br>◯ Waterways/Draper<br>◯ Downtown<br>◯ Timberlea<br>◯ Thickwood<br>◯ Wood Buffalo<br>◯ Parsons Creek<br>◯ Saprae Creek<br>◯ Gregoire/Saline Creek/Prairie Creek<br>◯ Other
	If other, please specify	__________________________________
6	Did you reside at Fort MacMurray during the 2020 flood?	◯ Yes<br>◯ No
	Where did you reside during the 2020 flood?	__________________________________
	Area of residence during the 2020 flood	◯ Abasand<br>◯ Beacon Hill<br>◯ Waterways/Draper<br>◯ Downtown<br>◯ Timberlea<br>◯ Thickwood<br>◯ Wood Buffalo<br>◯ Parsons Creek<br>◯ Saprae Creek<br>◯ Gregoire/Saline Creek/Prairie Creek<br>◯ Other

	If other, please specify	__________________________________
7	Where did you live prior to the 2016 Fort McMurray wildfire?	◯ Own home<br>◯ Renting<br>◯ Hostel<br>◯ Homeless<br>◯ Work camp
8	Where did you live prior to the 2016 Fort McMurray flooding?	◯ Own home<br>◯ Renting<br>◯ Hostel<br>◯ Homeless<br>◯ Work camp
9	Where do you live now?	◯ Own home<br>◯ Renting<br>◯ Hostel<br>◯ Homeless<br>◯ Work camp
10	Have you received a mental health diagnosis from a health professional? Please check all that apply.	☐ Depression<br>☐ Bipolar Disorder<br>☐ Anxiety<br>☐ Alcohol Abuse<br>☐ Drug Abuse<br>☐ Schizophrenia<br>☐ Personality Disorder<br>☐ Other diagnoses (not listed)<br>☐ I have never received a mental health diagnosis from a health professional
	If diagnosis not listed, please specify	__________________________________
11	Are you on any of the following medication for a mental health concern? Please check all that apply.	☐ Antidepressants<br>☐ Antipsychotics<br>☐ Benzodiazepines<br>☐ Mood Stabilizers<br>☐ Sleeping Tablets<br>☐ Other mental health medications not listed<br>☐ I am not on any medication for mental health concerns
16	If medication not listed, please specify:	__________________________________
12	Have you received mental health counselling in the past year?	◯ Yes<br>◯ No
13	Would you like to receive mental health counselling?	◯ Yes<br>◯ No
14	Have you used/abused alcohol or any drug/substance in the past year?	◯ Yes<br>◯ No

	Have you used/abused any of the following drugs in the past year? (Select all that apply)	☐ Downers or Sedatives (Barbiturates, etc.)<br>☐ Benzos (Valium, Xanax, etc.)<br>☐ Hallucinogens (including ecstasy, LSD, PCP, angle dust, mescaline, peyote, psilocybin, mushrooms)<br>☐ Alcohol<br>☐ Solvents/Inhalants (Thinners, gas, gasoline/petrol, glue, solution, trichlorethylene)<br>☐ Heroin or other Opiates (Morphine, etc.)<br>☐ Marijuana<br>☐ GHB<br>☐ Anabolic Steroids<br>☐ Stimulants (cocaine, amphetamine)<br>☐ Other
	If 'Other', please specify:	__________________________________
15	Have you received addiction counselling in the past year?	◯ Yes<br>◯ No
16	Would you like to receive addiction counselling?	◯ Yes<br>◯ No

Fort McMurray 2016 Wildfire

17	Where did you live on the 3rd of May when there was an order to evacuate Fort McMurray during the 2016 Wildfires?	◯ In Fort McMurray<br>◯ In Alberta but not in Fort McMurray<br>◯ In another province
18	Did you witness the burning of any homes or structures by the wildfires in Fort McMurray?	◯ Yes<br>◯ No
19	On the day of the evacuation, were you fearful for your life or the lives of your friends or family?	◯ Yes<br>◯ No
20	During the period of the evacuation order for Fort McMurray, how frequently did you watch television images about the devastation caused by the wildfires in Fort McMurray?	◯ Daily<br>◯ About every other day<br>◯ About once a week<br>◯ Less than once a week<br>◯ I did not watch the TV images of the devastation
21	During the period of the evacuation order for Fort McMurray, how frequently did you read newspaper and internet articles related to the devastation caused by the wildfires in Fort McMurray?	◯ Daily<br>◯ About every other day<br>◯ About once a week<br>◯ Less than once a week<br>◯ I did not read newspaper and internet articles related to the devastation
22	Did you lose property as a result of the wildfires in Fort McMurray? Please check all that apply	☐ Home was completely destroyed<br>☐ Home suffered substantial smoke damage<br>☐ Home suffered slight smoke damage<br>☐ Car was completely destroyed by the fire<br>☐ Business was completely destroyed by the fire<br>☐ Suffered no loss of property in the fire
23	Do you live in the same house you lived in before the evacuation order came into effect?	◯ Yes<br>◯ No; I live in a different house even though my previous home was not destroyed by the fire<br>◯ No; I live in a different house because my previous home was destroyed by the fire
24	Did you receive sufficient support from family and friends after the evacuation order for Fort McMurray was declared?	◯ Yes; I have had absolute support<br>◯ Yes; I have had some support<br>◯ Yes; but only limited support<br>◯ Not at all
25	Did you receive sufficient support from the Red Cross after the evacuation order for Fort McMurray was declared?	◯ Yes; I have had absolute support<br>◯ Yes; I have had some support<br>◯ Yes; but only limited support<br>◯ Not at all
26	Did you receive sufficient support from the Government of Alberta after the evacuation order for Fort McMurray was declared?	◯ Yes; I have had absolute support<br>◯ Yes; I have had some support<br>◯ Yes; but only limited support<br>◯ Not at all
27	Did you receive sufficient support from your insurers after the evacuation order for Fort McMurray was declared?	◯ Yes; I have had absolute support<br>◯ Yes; I have had some support<br>◯ Yes; but only limited support<br>◯ Not at all

28 Did you receive any counselling when you returned to Fort McMurray after the wildfires?

◯ Yes
◯ No

Fort McMurray 2020 Floods

29	Where did you live just prior to the 2020 Fort McMurray flooding?	◯ In Fort McMurray<br>◯ In Alberta but not in Fort McMurray<br>◯ In another province
30	Did you witness the flooding of homes or structures in Fort McMurray?	◯ Yes<br>◯ No
31	Were you fearful for your life or the lives of your friends or family during the flooding?	◯ Yes<br>◯ No
32	During the 2020 Fort McMurray flooding, how frequently did you watch television images about the devastation caused by the floods?	◯ Daily<br>◯ About every other day<br>◯ About once a week<br>◯ Less than once a week<br>◯ I did not watch the TV images of the devastation
33	During the 2020 Fort McMurray flooding how frequently did you read newspaper and internet articles related to the devastation caused by flooding?	◯ Daily<br>◯ About every other day<br>◯ About once a week<br>◯ Less than once a week<br>◯ I did not read newspaper and internet articles related to the devastation
34	Did you lose property as a result of the floods in Fort McMurray? Please check all that apply	☐ Home was completely destroyed<br>☐ Home suffered substantial damage<br>☐ Home suffered slight damage<br>☐ Car was completely destroyed by the floods<br>☐ Business was completely destroyed by the floods<br>☐ Suffered no loss of property in the floods
35	Do you live in the same house you lived in before the floods?	◯ Yes<br>◯ No; I live in a different house even though my previous home was not destroyed by the flood<br>◯ No; I live in a different house because my previous home was destroyed by the flood
36	Did you receive sufficient support from family and friends during and after the floods?	◯ Yes; I have had absolute support<br>◯ Yes; I have had some support<br>◯ Yes; but only limited support<br>◯ Not at all
37	Did you receive sufficient support from the Red Cross during and after the floods?	◯ Yes; I have had absolute support<br>◯ Yes; I have had some support<br>◯ Yes; but only limited support<br>◯ Not at all<br>◯ Not Applicable as I was not impacted by the floods
38	Did you receive sufficient support from the Government of Alberta during and after the floods?	◯ Yes; I have had absolute support<br>◯ Yes; I have had some support<br>◯ Yes; but only limited support<br>◯ Not at all<br>◯ Not Applicable as I was not impacted by the floods

39	Did you receive sufficient support from your insurers during and after the floods?	◯ Yes; I have had absolute support<br>◯ Yes; I have had some support<br>◯ Yes; but only limited support<br>◯ Not at all<br>◯ Not Applicable as I was not impacted by the floods

COVID-19 Mental Health Impacts

40 During the pandemic, have you been fearful about contracting the coronavirus?
◯ Yes
◯ No

41 During the pandemic, have you been fearful about your close friends or family memebrs contracting the coronavirus?
◯ Yes
◯ No

42 Have any of your close friends or family members been sick from the coronavirus disease?
◯ Yes
◯ No

43 Have you had to self-isolate or self-quarantine due to COVID-19 symptoms, recent travel, or because you were in contact with someone who may have COVID-19?
◯ Yes
◯ No

44 During the period of the COVID-19 pandemic, how frequently did you watch television images of sick and dead people caused by coronavirus?
◯ Daily
◯ About every other day
◯ About once a week
◯ Less than once a week
◯ I did not watch the TV images of the pandemic

45 During the period of the COVID-19 pandemic, how frequently did you read newspaper and internet articles related to the pandemic?
◯ Daily
◯ About every other day
◯ About once a week
◯ Less than once a week
◯ I did not read newspaper and internet articles related to the pandemic

46 Did you lose your job due to the COVID-19 pandemic?
◯ Yes
◯ No

47 Have you had sufficient support from family and friends since the COVID-19 pandemic declared?
◯ Yes; I have had absolute support
◯ Yes; I have had some support
◯ Yes; but only limited support
◯ Not at all

48 Have you had sufficient support from the Government of Canada since the COVID-19 pandemic declared?
◯ Yes; I have had absolute support
◯ Yes; I have had some support
◯ Yes; but only limited support
◯ Not at all

49 Have you had sufficient support from the Government of Alberta since the COVID-19 pandemic declared?
◯ Yes; I have had absolute support
◯ Yes; I have had some support
◯ Yes; but only limited support
◯ Not at all

50 Have you had sufficient support from your employer since the COVID-19 pandemic declared?
◯ Yes; I have had absolute support
◯ Yes; I have had some support
◯ Yes; but only limited support
◯ Not at all

For each of the following statements, please indicate your level of agreement

51 I tend to bounce back quickly after hard times.
- ◯ Strongly Agree
- ◯ Agree
- ◯ Neutral
- ◯ Disagree
- ◯ Strongly Disagree

52 I have a hard time making it through stressful events.
- ◯ Strongly Agree
- ◯ Agree
- ◯ Neutral
- ◯ Disagree
- ◯ Strongly Disagree

53 It does not take me long to recover from a stressful event.
- ◯ Strongly Agree
- ◯ Agree
- ◯ Neutral
- ◯ Disagree
- ◯ Strongly Disagree

54 It is hard for me to snap back when something bad happens.
- ◯ Strongly Agree
- ◯ Agree
- ◯ Neutral
- ◯ Disagree
- ◯ Strongly Disagree

55 I usually come through difficult times with little trouble.
- ◯ Strongly Agree
- ◯ Agree
- ◯ Neutral
- ◯ Disagree
- ◯ Strongly Disagree

56 I tend to take a long time to get over setbacks in my life
- ◯ Strongly Agree
- ◯ Agree
- ◯ Neutral
- ◯ Disagree
- ◯ Strongly Disagree

How often have you been bothered by the following problems over the last 2 weeks?

57	Little interest or pleasure in doing things.	◯ Not At All<br>◯ Several Days<br>◯ More Than Half the Days<br>◯ Nearly Everyday
58	Feeling down, depressed, or hopeless.	◯ Not At All<br>◯ Several Days<br>◯ More Than Half the Days<br>◯ Nearly Everyday
59	Trouble falling or staying asleep, or sleeping too much.	◯ Not At All<br>◯ Several Days<br>◯ More Than Half the Days<br>◯ Nearly Everyday
60	Feeling tired or having little energy.	◯ Not At All<br>◯ Several Days<br>◯ More Than Half the Days<br>◯ Nearly Everyday
61	Poor appetite or overeating.	◯ Not At All<br>◯ Several Days<br>◯ More Than Half the Days<br>◯ Nearly Everyday
62	Feeling bad about yourself - or that you are a failure or that you have let yourself or your family down.	◯ Not At All<br>◯ Several Days<br>◯ More Than Half the Days<br>◯ Nearly Everyday
63	Trouble concentrating on things, such as reading the newspaper or watching television.	◯ Not At All<br>◯ Several Days<br>◯ More Than Half the Days<br>◯ Nearly Everyday
64	Moving or speaking so slowly that other people could have noticed? Or the opposite - being so fidgety or restless that you have been moving around a lot more than usual.	◯ Not At All<br>◯ Several Days<br>◯ More Than Half the Days<br>◯ Nearly Everyday
65	Thoughts that you would be better off dead or of hurting yourself in some way.	◯ Not At All<br>◯ Several Days<br>◯ More Than Half the Days<br>◯ Nearly Everyday

How often have you been bothered by the following problems over the last 2 weeks?

66 Feeling nervous, anxious, or on edge.
- ◯ Not At All
- ◯ Several Days
- ◯ More Than Half the Days
- ◯ Nearly Everyday

67 Not being able to stop or control worrying.
- ◯ Not At All
- ◯ Several Days
- ◯ More Than Half the Days
- ◯ Nearly Everyday

68 Worrying too much about different things.
- ◯ Not At All
- ◯ Several Days
- ◯ More Than Half the Days
- ◯ Nearly Everyday

68 Trouble relaxing.
- ◯ Not At All
- ◯ Several Days
- ◯ More Than Half the Days
- ◯ Nearly Everyday

69 Being so restless that it's hard to sit still.
- ◯ Not At All
- ◯ Several Days
- ◯ More Than Half the Days
- ◯ Nearly Everyday

70 Becoming easily annoyed or irritable.
- ◯ Not At All
- ◯ Several Days
- ◯ More Than Half the Days
- ◯ Nearly Everyday

71 Feeling afraid as if something awful might happen.
- ◯ Not At All
- ◯ Several Days
- ◯ More Than Half the Days
- ◯ Nearly Everyday

Below is a list of problems and complaints people sometimes have in response to stressful life experiences. Please read each one carefully and choose the option that best matches how much you have been bothered by that problem in the LAST MONTH.

73	Repeated, disturbing memories, thoughts, or images of a stressful experience from the past?	◯ Not at All<br>◯ A Little Bit<br>◯ Moderately<br>◯ Quite A Bit<br>◯ Extremely
74	Repeated, disturbing dreams of a stressful experience from the past?	◯ Not at All<br>◯ A Little Bit<br>◯ Moderately<br>◯ Quite A Bit<br>◯ Extremely
75	Suddenly acting or feeling as if a stressful experience were happening again (as if you were reliving it)?	◯ Not at All<br>◯ A Little Bit<br>◯ Moderately<br>◯ Quite A Bit<br>◯ Extremely
76	Feeling very upset when something reminded you of a stressful experience from the past?	◯ Not at All<br>◯ A Little Bit<br>◯ Moderately<br>◯ Quite A Bit<br>◯ Extremely
77	Having physical reactions (e.g., heart pounding, trouble breathing, or sweating) when something reminded you of a stressful experience from the past?	◯ Not at All<br>◯ A Little Bit<br>◯ Moderately<br>◯ Quite A Bit<br>◯ Extremely
78	Avoid thinking about or talking about a stressful experience from the past or avoid having feelings related to it?	◯ Not at All<br>◯ A Little Bit<br>◯ Moderately<br>◯ Quite A Bit<br>◯ Extremely
79	Avoid activities or situations because they remind you of a stressful experience from the past?	◯ Not at All<br>◯ A Little Bit<br>◯ Moderately<br>◯ Quite A Bit<br>◯ Extremely
80	Trouble remembering important parts of a stressful experience from the past?	◯ Not at All<br>◯ A Little Bit<br>◯ Moderately<br>◯ Quite A Bit<br>◯ Extremely
81	Loss of interest in things that you used to enjoy?	◯ Not at All<br>◯ A Little Bit<br>◯ Moderately<br>◯ Quite A Bit<br>◯ Extremely

82 Feeling distant or cut off from other people?
◯ Not at All
◯ A Little Bit
◯ Moderately
◯ Quite A Bit
◯ Extremely

83 Feeling emotionally numb or being unable to have loving feelings for those close to you?
◯ Not at All
◯ A Little Bit
◯ Moderately
◯ Quite A Bit
◯ Extremely

84 Feeling as if your future will somehow be cut short?
◯ Not at All
◯ A Little Bit
◯ Moderately
◯ Quite A Bit
◯ Extremely

85 Trouble falling or staying asleep?
◯ Not at All
◯ A Little Bit
◯ Moderately
◯ Quite A Bit
◯ Extremely

86 Feeling irritable or having angry outbursts?
◯ Not at All
◯ A Little Bit
◯ Moderately
◯ Quite A Bit
◯ Extremely

87 Having difficulty concentrating?
◯ Not at All
◯ A Little Bit
◯ Moderately
◯ Quite A Bit
◯ Extremely

88 Being "super alert" or watchful on guard?
◯ Not at All
◯ A Little Bit
◯ Moderately
◯ Quite A Bit
◯ Extremely

89 Feeling jumpy or easily startled?
◯ Not at All
◯ A Little Bit
◯ Moderately
◯ Quite A Bit
◯ Extremely

FMM Strong daily supportive messaging program

Thank you for completing the survey questions.

If you will like to receive 12 months of free daily supportive text messages from the FMMStrong Supportive Text Messaging Program, please enter your phone number in the space provided.

____

The FMMStrong Supportive Text Messaging Program is a unique evidence-based e-mental health support developed by researchers at the University of Alberta to support residents of Fort McMurray on the fifth anniversary of the 2016 wildfires.

The daily messages help to address Stress, Anxiety, Depression and PTSD.

You can learn about the benefits of daily supportive text messaging from this link:
https://www.mdpi.com/1660-4601/18/4/2157
